# Relationships between Perceptual Attributes and Rheology in Over-the-Counter Vaginal Products: A Potential Tool for Microbicide Development

**DOI:** 10.1371/journal.pone.0105614

**Published:** 2014-09-04

**Authors:** Ellen D. Mahan, Toral Zaveri, Gregory R. Ziegler, John E. Hayes

**Affiliations:** 1 Sensory Evaluation Center, College of Agricultural Sciences, The Pennsylvania State University, University Park, Pennsylvania, United States of America; 2 Department of Food Science, College of Agricultural Sciences, The Pennsylvania State University, University Park, Pennsylvania, United States of America; University of Cape Town, South Africa

## Abstract

Vaginal microbicides are believed to have substantial potential to empower women to protect themselves from HIV, although clinical trials to date have had mixed results at best. Issues with patient adherence in these trials suggest additional emphasis should be placed on optimizing acceptability. Acceptability is driven, in part, by the sensory properties of the microbicide, so better understanding of the relationships between sensory properties and the physical and rheological properties of microbicides should facilitate the simultaneous optimization of sensory properties in parallel with the biophysical properties required for drug deployment. Recently, we have applied standard methods to assess the potential acceptability of microbicide prototypes ex vivo and to quantify the sensory properties of microbicide surrogates. Here, we link quantitative perceptual data to the rheological properties of 6 over-the counter (OTC) vaginal products used as ex vivo microbicide surrogates. Shear-thinning behavior (n) and tan δ (10 rad/s) showed no relationship with any perceptual attributes while shear storage modulus, G’ (10 rad/s) was correlated with some attributes, but did not appear to be a strong predictor of sensory properties. Conversely, the storage loss modulus, G” (10 rad/s) and the consistency coefficient, K, were correlated with several sensory attributes: stickiness, rubberiness, and uniform thickness for G’’ and stickiness, rubberiness, and peaking for K. Although these relationships merit confirmation in later studies, this pilot study suggests rheological principles can be used to understand the sensory properties evoked by microbicide surrogates assessed ex vivo. Additional work is needed to determine if these findings would apply for microbicides in vivo.

## Introduction

A microbicide is a product containing agents known to block HIV and other sexually transmitted diseases that can be inserted into the vagina prior to intercourse in the form of a gel, cream, foam, sponge, suppository, or film [1]. Currently, a number of microbicide products are in clinical trial [2], but there are no conclusive reports of effectiveness. Studies with different use protocols (coitally associated dosing versus daily use) have demonstrated varying levels of effectiveness in HIV prevention and this may be attributed to some extent to user adherence [3], [4]. Previously, work on microbicide development has proceeded in two parallel and generally independent tracks, as studies either focused on behavior or formulation but not both. In the former, focus groups and clinical trials were used to identify factors that influence user acceptability [5], [6], while the latter approach emphasized the way various physicochemical properties affect pharmacokinetics [7]. More recently, a stronger emphasis has been placed on efforts that combine these two approaches to study the interaction between physical properties and consumer acceptability [8]–[12]. However, attempts to link human perception (as defined by sensory or consumer studies) to instrumental data are complicated. Still, this approach appears to have good face validity, as many terms associated with the texture profile of a product are tied to rheological principles [13].

Recently, we adapted standardized methods from human sensory science to over-the-counter (OTC) vaginal products [14]. Although this was the first time these methods had been applied to commercially available microbicide surrogates, sensory specialists and product developers have used similar methods for decades to optimize product formulations. Specifically, quantitative descriptive profiling (descriptive analysis) has been utilized by the food and consumer products industries since the middle of the last century [15], [16]. These methods capitalize on the assumption that a group of individuals, often referred to as a panel, can be trained to describe and reliably quantify product perceptions. In order to be recruited into such descriptive panels, individuals must be able to detect small differences between products, describe these differences verbally and use scales accurately to quantify their observations. Once these criteria are met, the selected panelists go through an intensive training process, where a panel leader facilitates development of a lexicon the participants use to describe relevant attributes of the products. Concrete references with and without the property in question are provided to align panelists to the attribute concept, and reference stimuli are used to define a range of sensations along the continuum of the attribute. For example ‘peaking’ might be defined ‘as degree to which a product stands up when tapped’, and exemplified with mineral oil (none) and lanolin (a lot) [14]. After the training phase is complete, quantitative data are collected independently and blindly and then analyzed statistically. The goal is to get consistent and reliable quantitative data that describe perceptual attributes of a product [15]. Originally applied to food products, these methods have also been applied to non-food consumer goods like lotions and skin creams [17], [18]. Notably, descriptive panelists are never asked for hedonic or affective information about products [15], [16]; instead larger numbers of untrained consumers are used for affective data collection (e.g. [19], [20]).

Exploring the relationships between sensory and instrumental measures has been the focus of numerous studies (e.g. [21]–[23]). While many attempts are product specific, others have sought to create models using classical instrumental measurements [24], [25] or develop new instrumental evaluation techniques [26] that could be applied to predict sensory properties for a broad range of products. Replacing human assessors with instrumental measurements is advantageous as instrumental methods are less expensive and time consuming, and are often reproducible even when collected by instruments in different locations [27]. However, as with any instrumental method, these analytical tools may measure properties of a product that cannot be perceived by humans. Physical properties can also fall into this realm, a matter that has led Szczesniak [28], a seminal researcher in this area, to stress that only physical properties that are actually perceived by humans can fall under the definition of “texture”. Textural attributes are often multifaceted and evaluated using multiple senses including sight, touch, and sound [28]. Further, though some instrumental measurements can reliably predict sensory perception, extreme care must be taken to avoid ‘out of range’ problems that may result when a sensory system is saturated (i.e., at terminal threshold) [15], [27]. A simple example of this is hardness; glass and diamond have different hardness values when measured instrumentally, but are equally hard to the human finger. As such, finding an instrumental measurement, or group of instrumental measurements, to replace human assessor must be handled carefully.

Relationships between sensory and instrumental measures are usually defined using correlations; however, given the large number of attributes assessed during descriptive profiling, interpreting a large correlation matrix can become unmanagable. Multivariate data reduction techniques are commonly applied to simplify and analyze large data sets; forming factors allows data to be reduced and resulting plots easily visualize the relationships within and between products and attributes [15]. Previously, we used applied descriptive profiling methods to 6 OTC vaginal products in a panel of human assessors [14]. Here, rheological properties were measured, and Principal Components Analysis (PCA) was used to characterize sensory and rheological attributes separately on over-the-counter (OTC) vaginal products. Multiple Factor Analysis (MFA) was then used to integrate sensory (e.g. human) and rheological (e.g. instrumental) measures into a composite space. The behavioral data collection process and summary of results have been reported elsewhere [14].

## Materials

Three sexual lubricants (Astroglide, KY, PreSeed), two vaginal moisturizers (Replens, RepHresh), and one vaginal contraceptive (Gynol II) were used in this study. Replens and PreSeed were donated by a collaborator; all other products were purchased at a retail location in State College, PA.

## Methods

### Ethics Statement

Human methods and data were described previously [14]. Participants provided written informed consent and were reimbursed for their time. All procedures, including the consent process, were approved by the Pennsylvania State University Institutional Review Board (protocol #32606).

### Sensory Data

Perceptual evaluation of the 6 OTC vaginal products was performed ex vivo (in between fingers and on the forearm) and these were reported previously. Briefly, 36 variables rated in triplicate using quantitative descriptive profiling were evaluated by 10 individuals [14]. As in our previous report, only one measurement from each construct (thickness, slipperiness, graininess, etc) was used for analysis; however, the exact variables used in the present analyses differ from those used previously, as they were selected using a more sophisticated algorithm (described below in the statistics section). This is not expected to affect interpretation of the sensory data as within-construct variables were highly correlated (data not shown).

### Rheological Data

Instrumental data were collected in triplicate using an ARES rheometer (TA Instruments) at 25°C. Cone-and-plate geometry was used (0.04 radian cone angle, 50 mm diameter and 43 µm gap). A sample (1.5 ml) was loaded onto the plate and both oscillatory and steady-shear flow behavior were measured. Oscillatory measurements were collected using a dynamic frequency sweep conducted over the frequency range of 1–100 rad/s at 5% strain; preliminary testing determined that 5% strain produced a measureable torque within the linear viscoelastic range. Steady-shear flow behavior characterization followed. Viscosity was measured as a function of shear rate using a steady rate sweep from 1–100 s^−1^. Preliminary testing showed some samples to be thixotropic (viscosity decreased over time when a constant stress was applied) so all samples were subjected to a 300 s preshear at the corresponding shear rate (1–100 s^−1^). Temperature and shear rate for rheological analysis were selected to correspond to conditions similar to those used by the human assessors [14].

### Statistical Analyses

Multivariate data reduction techniques are a common approach in sensory science to simplify large data sets by restructuring them into a usable form based on patterns within products and attributes. Principal Components Analysis (PCA) and Multiple Factor Analysis (MFA) are multivariate techniques that are often applied to sensory datasets [15]. These techniques identify highly correlated variables within a data set and combine them into a new variable (factor); the first factor accounts for the maximum amount of variance within the data and additional uncorrelated factors are created from the remaining variance. The formation of factors allows data to be reduced to easily visualize the relationships within and between products and attributes [15]. PCA and MFA differ in that PCA groups together the variance of all attributes together while MFA allows the variance to be segmented by groups of attributes, for instance between a group of sensory attributes and a group of rheological attributes [29].

The *decat* (DEscription of CATegories) function in the SensoMineR package for the R statistical environment was used to determine which of the variables within each sensory construct were most discriminating among the product set [30]. The final attributes selected showed more significant differences among products and thus were more capable of distinguishing products in terms of their corresponding underlying sensory constructs.

Multiple Factor Analysis was conducted on the means of both the sensory and rheological data using the *MFA* function in the FactoMineR package for the R environment [31]. Kaiser’s criterion (eigenvalues >1) was used in determining the number of dimensions (principal components or factors) to retain [15]. When defining the significant attribute loadings for each dimension, the *dimdesc* function in SensoMineR was used with α = 0.1. Correlations among attributes, among rheological parameters, and between attributes and rheological parameters were calculated using StatPlus in Excel; correlations were considered significant at α = 0.05 and no adjustments were made for multiple comparisons, as the appropriate Bonferroni correction factor varies depending on the question of interest. Statistical analyses of the rheological data were performed in SAS 9.2 (Cary, NC) using a 2-way ANOVA (PROC GLM) followed by Tukey’s HSD for multiple comparisons. Significance was determined at α = 0.05.

## Results

### Sensory Data

A detailed presentation of the perceptual descriptive panel data from human assessors is provided elsewhere [14]. A brief summary follows. The contraceptive, Gynol, was thicker, stickier, grainier, more rubbery, had more air bubbles, was the least slippery, least smooth, and had the most uneven spread of all the samples. Gynol was the most perceptually different sample; this was confirmed by PCA (not reported). The two vaginal moisturizers, Replens and RepHresh, had very similar properties and only differed significantly from each other for the attributes stickiness and air bubbles (RepHresh is stickier while Replens has more air bubbles). Two lubricants, Pre-Seed and Astroglide, were very similar on several attributes; both products spread evenly, were very slippery (Astroglide >Pre-Seed), thin, smooth and clear and showed minimal peaking, air bubbles, stickiness, graininess, and rubberiness. The other lubricant, KY had more similarities with the moisturizers as it was thicker, stickier, more rubbery, and showed peaking and air bubbles; it also did not spread as evenly as Pre-Seed or Astroglide. However, KY was as slippery as Pre-Seed and was the only product besides Astroglide that was ropy. (Ropiness was defined as the ‘ability of the product to string between the finger and remaining product’; as discussed in [14], it could also be described as ‘snotty’ or ‘spinnbarkeit’).

In keeping with standard practices in the field, descriptive panelists are asked to rate the intensity of attributes using an analytical frame of mind following a lengthy training process; accordingly, no hedonic or acceptability data is collected from these panelists, as the attributes they have been trained to attend to and evaluate may or may not be drivers of acceptability in consumers. The resulting analytical data is then used to gain insight to affective/hedonic data collected separately from a group of naïve consumers who evaluate the product qualitatively or quantitatively.

### MFA

**Figure 1:**
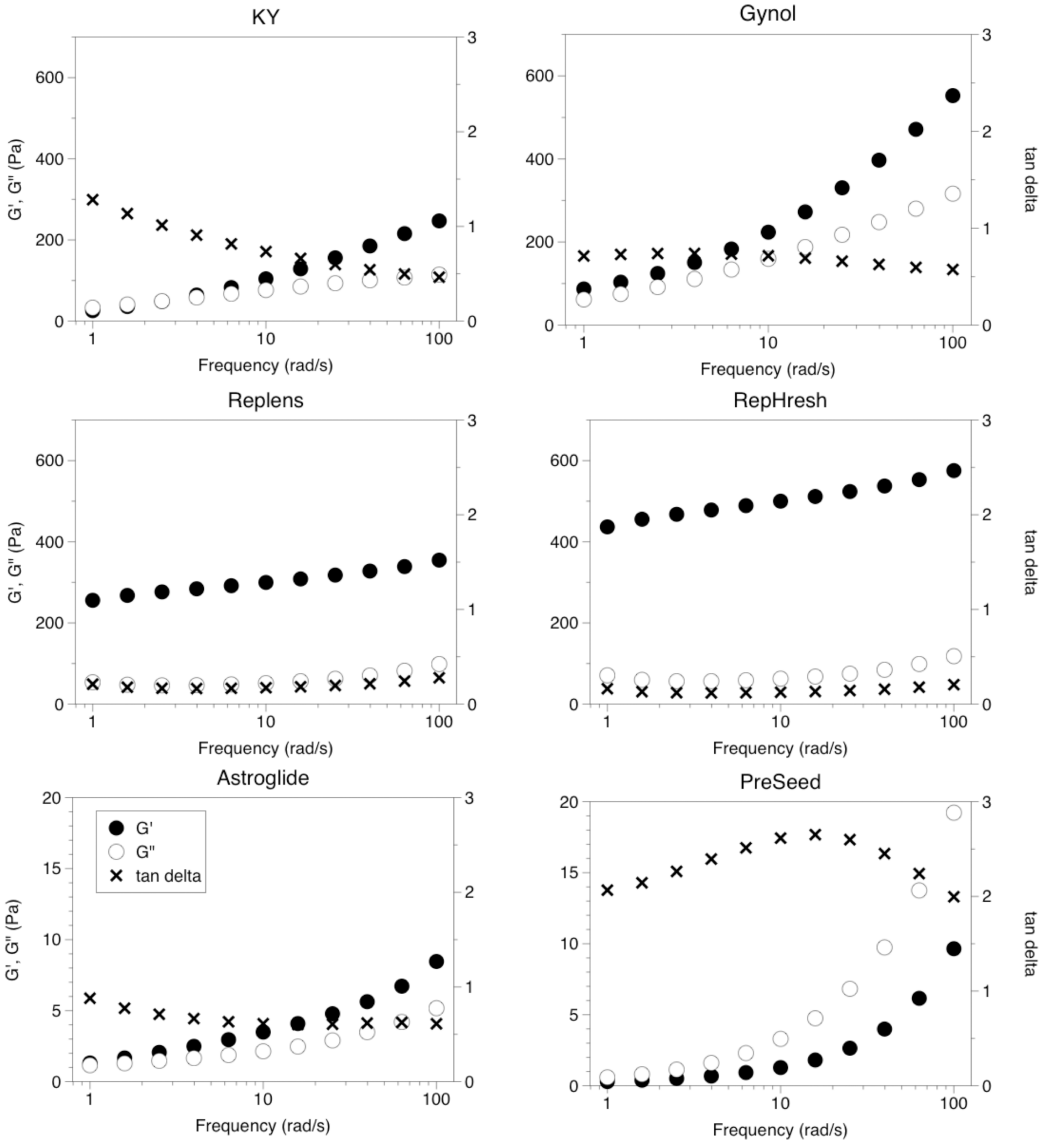
Multiple Factor Analysis plot of sensory and rheological data. Products are shown as squares. Rheological measures and sensory attributes are shown as vectors.

To examine the relationship between the rheological and sensory data on the sample set, MFA was performed using the sample means of both data sets (Figure 1). The first two factors were retained according to Kaiser’s criteria and these factors accounted for 84.1% of the total variance in the data set. Attribute loadings are displayed in Table 1. Factor 1 was positively correlated with the sensory attributes clumpiness, thickness, stickiness, peaking, rubberiness, and uniform thickness and with the rheological attributes G” and K; it was negatively correlated with the sensory attribute slipperiness. Factor 2 was positively correlated with the rheological attribute tan δ. Plotting of the attribute vectors onto the factor plot allows visualization of how attributes interact with samples (Figure 1). PreSeed was strongly influenced by the rheological attributes tan δ and n and by the sensory attribute slipperiness. Astroglide was distinguished primarily by the sensory attributes slipperiness, amount left, and ropiness. Gynol’s position was due to the sensory attributes graininess, air bubbles, clumpiness, uniform thickness, rubberiness, and stickiness as well as the rheological attributes G” and K. Replens and RepHresh were distinguished from each other primarily by their relative values of G’. KY did not have any variables that set it apart from the sample set and thus was positioned near the origin.

**Table 1:** MFA attribute loadings for sensory and rheological data.

Variable	PC1	PC2
Clumpiness	**0.807**	0.505
Thickness	**0.932**	−0.124
Slipperiness	**−0.909**	−0.152
Air Bubbles	0.701	0.555
Stickiness	**0.946**	0.094
Peaking	**0.978**	−0.096
Ropiness	−0.498	−0.436
Graniness	0.727	0.558
Rubberiness	**0.922**	0.115
Uniform Thickness	**0.916**	0.293
Amount Left	−0.585	−0.347
G′	0.725	−0.424
G″	**0.921**	0.314
Tan δ	−0.613	**0.755**
K	**0.976**	0.087
n	−0.667	0.711

Values in bold indicate variables were significantly correlated to the corresponding PC at α = 0.05. Significance was determined using the *dimdesc* function in R.

**Figure 2:**
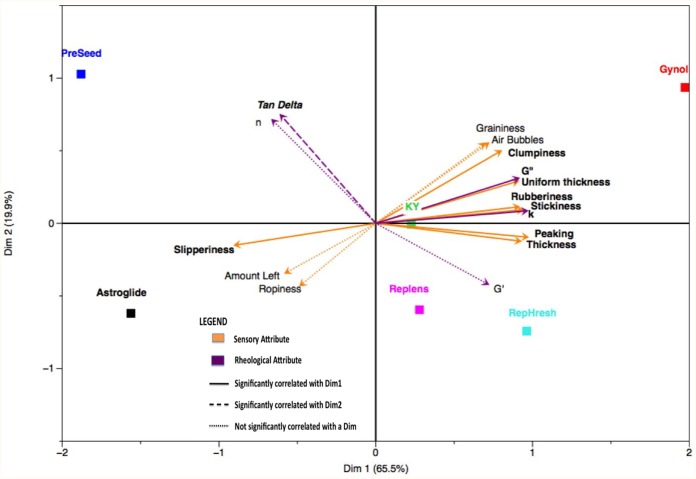
Individual correlations between G’’ and sensory attributes that loaded on PC1.

**Figure 3:**
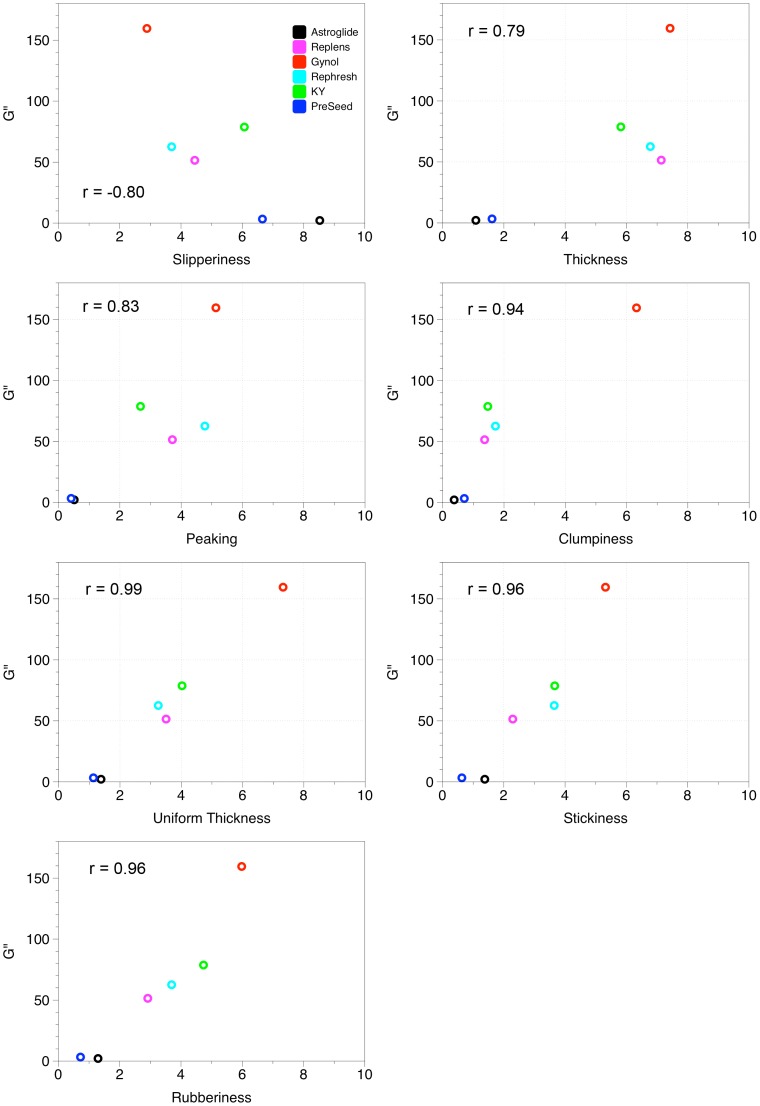
Individual correlations between K and sensory attributes that loaded on PC1.

To further explore interactions between sensory and rheological variables, correlation coefficients between attributes are listed in Table 2; correlations that were significant at α = 0.05 appear in bold. As seen in the positioning of the vectors in the MFA plot, tan δ and n were not significantly correlated with any of the sensory attributes. G’ was only significantly correlated with peaking. G” and K were significantly correlated with uniform thickness, stickiness, peaking, rubberiness, and clumpiness. G” was additionally correlated with graininess and air bubbles and K was correlated with thickness and slipperiness. Interpretations from the correlation coefficients should be considered cautiously, however, given the limited product space. Gynol, being high in many attributes and the only sample with attributes such as graininess and clumpiness, had a strong influence on the data set. The sample set influences correlations, such that the observed correlation between clumpiness and graininess may not be present, depending on the samples included. Also, sensory attributes that are highly correlated, such as peaking and thickness, may be redundant variables that measure the same underlying sensory construct [15]. Correlation graphs between G” and the PC1 correlated sensory attributes are shown in Figure 2 and between K and the PC1 correlated attributes are presented in Figure 3. Although correlation coefficients for all relationships were high (≥0.79) consideration of the plotted data again shows the strong influence of Gynol; Gynol had high values in many sensory attributes as well as G” and K and the influence of this product on the proposed perceptual-rheological relationships is visualized in the correlation plots. However, some relationships appeared to show perceptual properties could be explained by rheological factors, specifically the relationships between G” and uniform thickness, rubberiness, and stickiness and between K and peaking, rubberiness, and stickiness.

**Table 2:** Correlation matrix for sensory and rheological measures.

		Thickness	Graininess	UniformThickness	Stickiness	Peaking	Ropiness	Rubberiness	Clumpiness	Air Bubbles	Slipperiness	Amount Left	G'	G"	Tan δ	k	n
Thickness	Correlation Coefficient	1.00															
	p value																
Graininess	Correlation Coefficient	0.50	1.00														
	p value	0.312															
UniformThickness	Correlation Coefficient	0.80	**0.90**	1.00													
	p value	0.057	**0.014**														
Stickiness	Correlation Coefficient	0.82	0.78	**0.94**	1.00												
	p value	0.047	0.067	**0.006**													
Peaking	Correlation Coefficient	**0.95**	0.61	**0.82**	**0.87**	1.00											
	p value	**0.003**	0.201	**0.045**	**0.024**												
Ropiness	Correlation Coefficient	−0.61	−0.35	−0.43	−0.33	−0.57	1.00										
	p value	0.195	0.492	0.390	0.521	0.236											
Rubberiness	Correlation Coefficient	**0.84**	0.75	**0.94**	**0.98**	**0.84**	−0.35	1.00									
	p value	**0.037**	0.087	**0.005**	**0.000**	**0.037**	0.493										
Clumpiness	Correlation Coefficient	0.61	**0.99**	**0.94**	**0.83**	0.71	−0.44	0.80	1.00								
	p value	0.202	**0.000**	**0.006**	**0.039**	0.116	0.383	0.055									
Air Bubbles	Correlation Coefficient	0.49	**0.99**	**0.89**	0.74	0.58	−0.34	0.71	**0.98**	1.00							
	p value	0.329	**0.000**	**0.018**	0.094	0.227	0.507	0.112	**0.001**								
Slipperiness	Correlation Coefficient	**−0.90**	−0.64	−0.79	−0.77	**−0.95**	0.79	−0.74	−0.74	−0.62	1.00						
	p value	**0.013**	0.171	0.063	0.073	**0.004**	0.059	0.091	0.094	0.192							
Amount Left	Correlation Coefficient	−0.69	−0.37	−0.48	−0.41	–0.67	**0.99**	–0.42	–0.47	–0.36	**0.86**	1.00					
	p value	0.132	0.465	0.337	0.417	0.148	**0.000**	0.412	0.345	0.489	**0.028**						
G'	Correlation Coefficient	0.78	0.15	0.39	0.54	**0.85**	–0.50	0.47	0.28	0.11	–0.79	–0.62	1.00				
	p value	0.065	0.777	0.440	0.269	**0.034**	0.309	0.346	0.596	0.830	0.063	0.190					
G"	Correlation Coefficient	0.79	**0.90**	**0.99**	**0.96**	**0.83**	–0.45	**0.96**	**0.94**	**0.87**	–0.80	–0.50	0.41	1.00			
	p value	0.059	**0.016**	**0.000**	**0.003**	**0.041**	0.368	**0.002**	**0.006**	**0.023**	0.057	0.312	0.416				
Tan δ	Correlation Coefficient	–0.62	–0.11	–0.40	–0.53	–0.64	–0.16	–0.50	–0.18	–0.11	0.37	–0.04	–0.66	–0.37	1.00		
	p value	0.185	0.836	0.436	0.277	0.173	0.760	0.309	0.737	0.836	0.465	0.933	0.157	0.474			
k	Correlation Coefficient	**0.87**	0.75	**0.89**	**0.96**	**0.95**	–0.50	**0.92**	**0.82**	0.70	**–0.89**	–0.59	0.70	**0.92**	–0.51	1.00	
	p value	**0.026**	0.087	**0.016**	**0.002**	**0.004**	0.316	**0.009**	**0.044**	0.121	**0.016**	0.221	0.118	**0.009**	0.299		
n	Correlation Coefficient	–0.69	–0.15	–0.46	–0.59	–0.68	–0.10	–0.58	–0.22	–0.15	0.43	0.01	–0.67	–0.44	**0.99**	–0.57	1.00
	p value	0.129	0.775	0.354	0.213	0.134	0.852	0.228	0.67	0.779	0.398	0.978	0.147	0.387	**0.000**	0.241	

### Rheology Data

Most microbicidal vaginal “gels” described to date are not in fact gels in the strict rheological sense; rather, they are thick viscoelastic liquids or semi-solid creams. Viscoelastic materials are those intermediate between viscous liquids and elastic solids, and their properties are often measured using oscillatory rheological techniques. In these methods, the rheological response is deconvoluted into the relative elastic or solid component (characterized by the storage modulus, G’) and the viscous or liquid component (characterized by the loss modulus, G”). The ratio of the two is quantified as the tangent of the phase shift (phase angle), called tan δ, and directly related to the ratio of the energy lost to the energy stored per oscillation. Values for Tan δ can vary from 0 to infinity, and the closer Tan δ is to 0, the more solid the material’s character.

**Figure 4:**
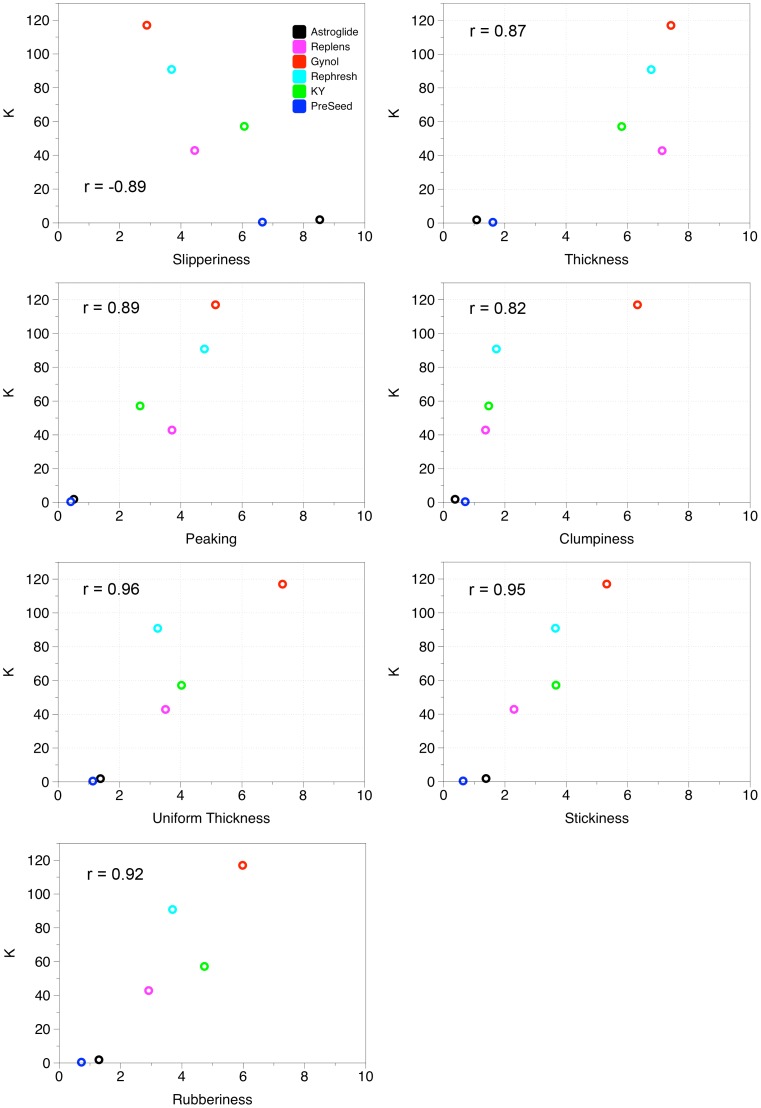
Shear storage modulus, G’, loss modulus, G” and tangent delta of samples over the frequency range 1–100 rad/s at 5% strain. Within each plot, G’ and G’’ are plotted on the left y-axis and tan delta is plotted on the right y-axis. To provide better resolution, the range differs for the left y-axis for the bottom two figures.

The results of the oscillatory frequency sweep can be seen in Figure 4; data at 10 rad/s was formally tested via ANOVA (Table 3). Tan δ (again, the ratio of energy lost (G”) to the energy stored (G’)) differed significantly among the samples (F (5,17) = 220.3, p<0.001).

**Table 3:** Oscillatory measurements with 5% strain at 10 rad/s.

Sample	G' (Pa)	G'' (Pa)	Tan delta
Astroglide	3.5**E**	2.1**E**	0.61**B**
Replens	298.5**B**	51.5**D**	0.17**C**
Gynol	223.7**C**	159.5**A**	0.72**B**
RepHresh	500.1**A**	62.6**C**	0.13**C**
KY	107.2**D**	78.7**B**	0.73**B**
PreSeed	1.3**E**	3.3**E**	2.62**A**

Means with different letters within columns are significantly different α = 0.05. Significance was determined using ANOVA followed by Tukey’s HSD in SAS.

PreSeed had the greatest tan δ value (tan δ>1), indicating a viscous liquid [32], [33]. Astroglide, Gynol, and KY did not differ significantly from one another and their tan δ values (tan δ<1) fell in the range of a viscoelastic solid while Replens and RepHresh, which did not differ significantly from one another, had the lowest tan δ values, implying they had the most solid-like character.

In terms of relative magnitude of G’ and G”, PreSeed and Astroglide were easily distinguished; G’ (F (5,17) = 944.4, p<0.001) and G” (F (5,17) = 634.7, p<0.001) differed significantly across the samples. At 10 rad/s, the G’ and G” of PreSeed and Astroglide are significantly lower than the other samples. Coinciding with tan δ values, RepHresh, followed by Replens, had the highest G’ values, while Gynol, followed by KY, had the highest G” values.

Flow behavior data was analyzed using the Ostwald-de Waele power law equation: η = Kγ^n–1^ with η as shear viscosity, K as consistency coefficient, γ as shear rate, and n as the power law index; all R^2^ values were greater than 0.99, indicating the data fit this model very well. The data are presented in Table 4. All samples showed pseudoplastic behavior (n<1); however, the degree of pseudoplasticity differed significantly across samples (F (5,17) = 1822.2, p<0.001). PreSeed was least shear thinning with a power law index close to 1 (n = 0.846); all other samples had power law coefficients between 0.36–0.197, with Replens and RepHresh being the most shear thinning.

**Table 4:** Flow behavior measurements.

Sample	K (Pa•s^n^)	n	R^2^
Astroglide	1.9**D^1^**	0.36**B**	1.000
Replens	42.8**C**	0.20**D**	0.993
Gynol	117.0**A**	0.33**C**	0.996
RepHresh	90.9**B**	0.20**D**	0.998
KY	57.1**C**	0.31**C**	0.997
PreSeed	0.5**D**	0.85**A**	0.994

Means with different letters within columns are significantly different α = 0.05. Significance was determined using ANOVA followed by Tukey’s HSD in SAS.

The consistency coefficient (K) also differed significantly among samples (F (5,17) = 209.9, p<0.001). The consistency coefficient of Gynol was the greatest, followed by RepHresh, then Replens and KY. Astroglide and PreSeed had the lowest consistency coefficients and did not differ significantly from each other.

The results discussed here, which were confirmed by Principal Components Analysis (PCA) (not reported), indicate that PreSeed was the most rheologically different sample as it displayed predominantly viscous liquid behavior and was the least shear thinning.

## Discussion

Textural attributes are a common focus of participants involved in microbicide acceptability studies. Terms such as “slippery”, “thick”, and “sticky” are often used by focus group participants to describe potential and surrogate microbicide products [34]–[36]; these attributes can be positive or negative, depending on the individual. Therefore, being able to predict the level of these attributes in a candidate microbicide during preclinical formulation may help predict its future success in the field. The use of quantitative descriptive profiling in this study, which provides objective quantitative measurements of perceptual attributes such as slipperiness and thickness, can provide a crucial link between focus groups and instrumental measures. Through this type of quantitative data, we can gain additional insight on the relationships between human perception and physical properties of microbicides and microbicide surrogates.

Correlations indicate whether an instrumental variable potentially predicts a sensory/perceptual attribute. This study found significant correlations between the perception of graininess, uniform thickness, stickiness, peaking, rubberiness, clumpiness, and air bubbles and the rheological attributes G” and K; peaking was also correlated with G’ and K was correlated with thickness and slipperiness. However, Szczesniak [37] cautions that such correlations are highly subject to the range and number of samples evaluated. Given the small product set evaluated in this pilot study, correlations reported here should be treated cautiously. Many significant correlations did not appear to show a relationship between the sensory attribute and rheological measure (i.e. correlation between slipperiness and K); additionally, some of the other rheological-sensory relationships may have been strongly influenced by Gynol, which had high values of many sensory attributes and rheological attributes.

Szczesniak also points out that the strength of correlations is subject to how well instrumental measurements imitate sensory evaluation conditions [37]. In this study, instrumental measurements were taken at standard room temperature, 25°C, since sensory evaluation samples were dispensed at room temperature; it is possible that contact with skin could have increased the temperature of these samples, changing their rheological properties. Oscillatory measurements were reported at 10 rad/s, which is roughly similar to the rate at which panelists manipulated samples (2 rotations/s); how well these frequencies align and influence oscillatory measurements should be considered. Often, instrumental measures are collected using novel instruments or techniques that mimic sensory evaluation [15], [38]. The measurements we selected here were based on measurements being used by other microbicide researchers to characterize the physical nature of potential microbicides [39]–[41].

In future studies, limitations of this initial proof of concept study should be addressed. Increasing the size and diversity of samples within the product space should eliminate any undue leverage of individual products on perceptual-rheological correlations. To better understand how rheological variables influence sensory perception of microbicides, the samples could be rationally designed to cover the complete rheological design space, which would reduce confounding variables introduced by using OTC products. Relating sensory properties to rationally designed samples would better demonstrate putatively causal relationships; these data could then used to build more complex regression models to predict sensory properties through the rheological variables.

Further, the rheological data we collected was by no means exhaustive; our parameters were chosen based on microbicide studies already being conducted and to gain an understanding of the physical structure of the products. However, when relating sensory attributes to instrumental data one must consider the multifaceted nature of human perception. Increasing the range and variety of rheological variables studied (including the measurements of G’, G”, and tan δ at different frequencies) will give a better perspective on physical structure of samples and any relationship with textural properties. Expanding the physical analyses beyond rheology may also provide more insight; approaches such as tribology, which measures a sample’s interaction with various surfaces (skin, glass, mucosa, etc), as well as yield stress which measures plastic deformation of the sample may be able to predict various attributes and enhance understanding of the perceptual properties. A recent study by our group shows how elastic properties of semisoft suppositories influences women’s perceived ease of insertion and willingness to try [42].

Addressing the issues with the sample set, rheological variables, and profiling procedure may help clarify relationships between the human and instrumental data. Some difficulty is introduced through the complex nature of the perceptual attributes, which could be redundant (meaning separately measured attributes are actually measuring the same underlying sensory construct) or too complex to be summarized by a single instrumental measurement. The improvements suggested above, including rationally designing samples and increasing rheological measurements, would enable a better demonstration of any relationships between the data; these data sets could further be used to build regression models that predict sensory perception through the rheological variables. However, useable information can still be gained, especially if this analysis is considered as a proof of concept study. Common rheological measures, shear-thinning behavior (n) and tan δ at 10 rad/s, showed no relationship with any textural attributes measured by the human participants; G’ at 10 rad/s was correlated with some attributes but did not appear to be a strong predictor of these attributes. G” at 10 rad/s and K were correlated with several sensory attributes; still, these correlations merit confirmation in later studies. The relationships between G” and stickiness, rubberiness, and uniform thickness and between K and stickiness, rubberiness, and peaking are more promising and suggest that these viscosity related measurements predict the perceptual attributes.

G” at a given frequency is a measure of dynamic viscosity [43] while K is related to the viscosity. As both these parameters measure viscosity in some form, it could explain their relationship with stickiness, uniform thickness, and peaking; for instance, a material with a high resistance to flow (viscosity) would require a great amount of force to separate the fingers after the sample had been compressed (stickiness). Beyond the obviously Gynol-influenced correlations with clumpiness, graininess, and air bubbles, the remaining perceptual-rheological correlations seem to point to an overall separation of the samples into two groups: a ‘low structure’ group (Astroglide and PreSeed), and a ‘high structure’ group (Replens, RepHresh, KY, and Gynol). Samples with positive PC1 values (‘high structure’) on the PCA and MFA plots were high in attributes G’, G”, K, uniform thickness, stickiness, peaking, thickness, and rubberiness while those to the left (‘low structure’) were low in those attributes. This may be due to a greater concentration of rheologically relevant materials in the ‘high structure’ group that allowed formation of structures within the sample that influence texture.

Recently, microbicide research has been shifting to try to explain user acceptability through biophysical properties [11]. Though this type of research is still in infancy, the studies that have been conducted already show how insight from descriptive profiling approaches can contribute and help inform study designs. A study by Verguet and colleagues, assessed women’s preferences for various OTC vaginal products including KY [8]. In their study, the samples were pre-qualified by the researchers as more or less ‘slippery’ and ‘thick’ based on the viscosity of the samples; participants were asked how they would potentially use these products based on the attributes specified by the researchers [8]. The goal of the Verguet study was only to assess women’s potential preferences and behaviors and were not actually tested *in vivo*, so data was not completely dependent on user perceptions of the slipperiness or thickness of the samples. Likewise, van der Berg and colleagues [44] characterized Pre-seed, KY, and Replens rheologically as part of an in mano acceptability study, stating that viscosity “corresponds to the colloquial notion of thickness”. They also provide instrumental data that Preseed had a lower viscosity than KY or Replens. Similarly, we previously generated quantitative perceptual data showing PreSeed is perceived as less thick than KY or Replens on the forearm [14]. Here, we extend those data, showing that perceptual attributes like thickness correspond to rheological measures like G’’ (the loss modulus) and K (the consistency coefficient) (see Figure 2). We also find that PreSeed and Replens differ greatly in their sheer thinning behavior; it is unknown how properties like sheer thinning influence user acceptability either in mano, or peri-coitally, but this would seem to be a fruitful area for future research. Given present results, we would recommend that future microbicide research should systematically consider how well physical measurements relate to the specific perceptual attributes they want to explore.

## Conclusions

Quantitative descriptive profiling, through its use of humans as calibrated sensors, can help close the gap between instrumental data and consumer acceptability (e.g. [19], [20]). By determining the ranges in which humans can perceive and differentiate rheological properties among samples, the process of relating sensory and instrumental data can help eliminate erroneous assumptions made when instrumental data are directly linked to acceptability data. Although present data are subject to the range of samples and instrumental methods selected, it demonstrates how descriptive profiling and rheological data of vaginal products can be integrated. Certainly, we do not mean to imply that sensations in the hand are a direct surrogate for sensations in the vagina, but rejection (and thus non-use) may arise from sensory properties well before a product is ever taken into the body. For example, an individual may reject natto (fermented soybeans) solely on the basis of their slimy texture without ever putting them in her mouth. Accordingly, the approach described here may provide new insights on perceptual-rheological relationships, as well as helping to rapidly eliminate unacceptable prototypes early in preclinical testing. Here, both sensory and instrumental measures appeared to discriminate between samples that had a high degree of internal structure from samples with a low degree of internal structure; however, separation of samples within each group are caused by variations in both sensory and rheological properties for individual samples. This analysis should inform those working in rational design of microbicides, and highlight the need to examine any instrumental measurements before attempting to relate them to human perception or acceptability.
